# Long-Lived Immunity in SARS-CoV-2-Recovered Children and Its Neutralizing Capacity Against Omicron

**DOI:** 10.3389/fimmu.2022.882456

**Published:** 2022-05-17

**Authors:** Justyna Sieber, Margareta Mayer, Klara Schmidthaler, Sonja Kopanja, Jeremy V. Camp, Amelie Popovitsch, Varsha Dwivedi, Jakub Hoz, Anja Schoof, Lukas Weseslindtner, Zsolt Szépfalusi, Karin Stiasny, Judith H. Aberle

**Affiliations:** ^1^ Division of Pediatric Pulmonology, Allergy and Endocrinology, Department of Pediatrics and Adolescent Medicine, Comprehensive Center of Pediatrics, Medical University of Vienna, Vienna, Austria; ^2^ Department of Clinical Immunology, Wroclaw Medical University, Wroclaw, Poland; ^3^ Center for Virology, Medical University of Vienna, Vienna, Austria

**Keywords:** COVID-19, neutralization, omicron, immune memory, SARS-CoV-2-specific T cells, vaccine, children

## Abstract

SARS-CoV-2 infection is effectively controlled by humoral and cellular immune responses. However, the durability of immunity in children as well as the ability to neutralize variants of concern are unclear. Here, we assessed T cell and antibody responses in a longitudinal cohort of children after asymptomatic or mild COVID-19 over a 12-month period. Antigen-specific CD4 T cells remained stable over time, while CD8 T cells declined. SARS-CoV-2 infection induced long-lived neutralizing antibodies against ancestral SARS-CoV-2 (D614G isolate), but with poor cross-neutralization of omicron. Importantly, recall responses to vaccination in children with pre-existing immunity yielded neutralizing antibody activities against D614G and omicron BA.1 and BA.2 variants that were 3.9-fold, 9.9-fold and 14-fold higher than primary vaccine responses in seronegative children. Together, our findings demonstrate that SARS-CoV-2 infection in children induces robust memory T cells and antibodies that persist for more than 12 months, but lack neutralizing activity against omicron. Vaccination of pre-immune children, however, substantially improves the omicron-neutralizing capacity.

## Introduction

Children exhibit mostly asymptomatic or mild COVID-19 and are less likely to experience severe respiratory disease than adults, but can develop MIS-C (multi-inflammatory syndromes) and long-term complications, such as long COVID (1–7). The mechanisms for the age-related differences in the clinical outcome are still under investigation, but hypotheses include differential expression of the virus entry receptor as well as differences in the functioning and strength of immune responses in children compared to adults (8–15). Previous studies indicated that children are capable of mounting a robust T cell and antibody response to SARS-CoV-2 infection (8, 10, 13, 14). Antibodies specific for the spike protein, which binds to the receptor and mediates virus entry, can exhibit potent neutralizing activity, and are a correlate of protection against COVID-19 (16–18). However, data about the durability of these antibody responses in children are limited (8, 10, 13), reporting reduced antibody breadth and neutralizing activity (10) as well as similar neutralizing capacity (8) in comparison to adults. An increasing rate of reinfections associated with the emergence of the omicron variant (sublineage BA.1) has also raised concerns about evasion of immunity induced by prior infection (19–21). More recently, there has been an upsurge of BA.2 sublineage, which has been spreading rapidly in Europe and Asia, accounting for 34.2% of omicron variant sequences detected at the beginning of March, 2022 (22). In comparison to previous variants of concern (VOCs), omicron variant sequences have a greater number of mutations in the spike protein, affecting critical epitopes in the receptor-binding domain (RBD) as well as in the N-terminal domain, that have been associated with resistance to neutralizing antibodies generated by prior infection or vaccination (23) and reviewed in (24). It is therefore important to improve our understanding of the durability of immune memory and how this immunity confers protection against symptomatic VOC infections.

Furthermore, the United States and many European countries have started their vaccination program for children (25, 26). The effect of vaccination in children with pre-existing immunity, however, remains poorly understood. Specifically, it is unclear how the immune response in recovered children may benefit from vaccination and whether vaccination has any effect on the quality of infection-induced responses, in particular with respect to the omicron variant.

Here, we addressed these questions by evaluating SARS-CoV-2-specific T cell and antibody responses in a longitudinal cohort of recovered children, including a comparison of neutralization of the omicron variant relative to an ancestral SARS-CoV-2 strain (D614G virus isolate from the early phase of the pandemic). Additionally, we evaluated recall responses of preexisting immunity with BNT162b2 (BioNTech-Pfizer) or Ad26.COV2-S (Johnson and Johnson) in a subgroup of SARS-CoV-2-recovered children compared with seronegative children generating a primary response to BNT162b2 vaccination. These studies provide insights into the durability of immune memory after SARS-CoV-2 infection in children and the benefits of boosting infection-induced immunity by vaccination.

## Materials and Methods

### Study Population

The study cohort was followed from the early phase of the pandemic (May-July 2020), before the emergence of SARS-CoV-2 VOCs alpha, beta, gamma, delta and omicron. All seropositive children had participated in a population-based SARS-CoV-2 seroprevalence study (27). These children and their families were followed up for 15 months. Serology and personal interviews with parents and children allowed classification of asymptomatic or mild infection. In the present study, 50 children were included to investigate whether asymptomatic or mild SARS-CoV-2 infection confers specific antibody and T cell responses lasting for 15 months. Of the 50 children, 26 were seropositive by SARS-CoV-2 RBD antibody testing and 24 were consistently seronegative throughout the follow up.

### Ethics Statement

All work was conducted in accordance with the Declaration of Helsinki in terms of informed consent and approval by an appropriate institutional board. Blood samples were obtained after donors and their parents consented to participate in this study. The ethics committee of the Medical University of Vienna, Austria, approved the study protocol (approval no. 2104/2020).

### Preparation of Blood Samples

PBMCs were isolated from blood samples using Ficoll-Paque Plus™ (GE Healthcare) and cryopreserved in liquid nitrogen. Serum samples were stored at −20°C.

### Detection of SARS‐CoV‐2‐Specific IgG

SARS‐CoV‐2‐specific antibodies were quantified using the Wantai SARS-CoV-2 IgG ELISA (Quantitative) kit (Beijing Wantai Biological Pharmacy Enterprise) as described previously (27). The assay is calibrated according to the WHO International standard for SARS-CoV-2 immunoglobulin, enabling the quantification of antibody concentrations in BAU/ml. Results were interpreted using a cut-off of 5.4 BAU/ml, as recommended by the manufacturer. The SARS-CoV-2 antibody profile against spike RBD, S1, S2 and nucleocapsid was assessed using the SARS-CoV-2 ViraChip assay (Viramed, Viramed Biotech Ag, Germany) as described previously (28). The SARS-CoV-2 ViraChip assay is calibrated according to the WHO International standard for SARS-CoV-2 immunoglobulin, enabling the quantification of antibody concentrations in BAU/ml. Results were interpreted using the following cut-offs, as recommended by the manufacturer: RBD≥15 BAU/ml; S1≥25 BAU/ml; S2≥72 BAU/ml; N≥16 BAU/ml.

### Viruses and Neutralization Test

The preparation of the D614G virus stock (GISAID accession number: EPI_ISL_438123) has been described previously (29). Following the same protocol, omicron BA.1 (GISAID accession number: EPI_ISL_9110894) and BA.2 (GISAID accession number: EPI_ISL_11110193) were isolated from nasopharyngeal swabs from COVID-19 patients, passaged on Vero E6-TMPRSS2 cells (kindly provided by Anna Repic), and subjected to next-generation sequencing. The isolates were controlled to be free of other respiratory viruses by PCR as described in Koblischke et al, 2020 (29) and were tested negative for mycoplasma with the MycoAlertTM Mycoplasma Detection Kit (Lonza Group Ltd, Basel, Switzerland).

The neutralization assay was carried out essentially as described previously (29). Briefly, serial dilutions of serum samples were incubated with 50-100 TCID50 SARS-CoV-2 for 1 hour at 37°C. The mixtures were then transferred to Vero E6 cells (ECACC 85020206) and incubation was continued for three (D614G) or five (omicron) days. NT titers were expressed as the reciprocal of the serum dilution required for protection against virus-induced cytopathic effects. NT titers ≥10 were considered positive.

### Peptides

For T cell stimulation, PepMixTM SARS-CoV-2 peptide pools (product codes: PM-WCPV-VEMP, PM-WCPV-VME, PM-WCPV-S, PM-WCPV-NCAP, and PM-SARS2-SMUT08-1) were purchased from JPT (Berlin, Germany). The pools comprise 15mer peptides overlapping by 11 amino acids and cover the entire sequences of the SARS-CoV-2 wildtype structural proteins: envelope (E), membrane (M), spike (S) composed of two sub-pools S1 (aa 1-643) and S2 (aa 633-1273), and nucleoprotein (N), and S from the omicron variant B.1.1.529. Peptides were dissolved in dimethyl sulfoxide and diluted in AIM-V medium for use in PBMC expansion and intracellular cytokine staining (ICS) assays.

### Flow Cytometry Staining Following 10 Day *In Vitro* Stimulation

*In vitro* expansion of PBMCs was performed as described previously (30). In brief, cryopreserved PBMCs were thawed in pre-warmed StableCell RPMI-1640 medium (Sigma) containing 10% FBS (FBS 12-A, Capricorn), 10 mM Hepes (Sigma), 50 IU/ml Pen-Strep (Sigma) and 50 IU IL-2 (Peprotech) at a concentration of 1×10^6^ cells/ml. PBMCs were pulsed with peptides (1 μg/ml) and cultured for 10 days adding 100 IU IL-2 on day 5. *In vitro* expanded cells were analyzed by intracellular cytokine and cell surface marker staining. PBMCs were incubated with 2 μg/ml of peptide and 1 μg/ml anti-CD28/49d antibodies (L293 and L25, Becton Dickinson) or with anti-CD28/49d antibodies alone (negative control) for 6h. After 2h, 0.01 μg/ml brefeldin A (Sigma) was added. Staining was performed using APC/H7 anti-human CD3 (SK7, Becton Dickinson), Pacific Blue anti-human CD4 (RPA-T4, Becton Dickinson), PE anti-human CD8 (HIT8a, Becton Dickinson), FITC anti-human IFN-γ (25723.11, Becton Dickinson), and Fix/Perm kit (Invitrogen). Viable cells were determined using Live/Dead Fixable Aqua Dead (Invitrogen). The gates for detection of cytokines in peptide-stimulated cell samples were set in the samples with no antigen stimulation. Antigen-specific responses were determined as the frequency of IFN-γ positive T cells in stimulated samples with background subtraction from paired unstimulated controls (30). The cut-off was determined by background staining (no peptide) of all negative controls (31). For all experiments combined, the mean background was 0.44%, 0.16% and 0.26% for samples expanded with spike (composed of subpools S1 and S2), membrane or nucleocapsid peptide pools, respectively. To exclude non-specific or background responses, responses >3-fold above the background of the respective peptide pools were considered positive.

### Statistical Analysis

Subject characteristics were described by medians and interquartile ranges (IQR) for continuous variables and frequencies and percentages for categorical variables. Statistical analysis was performed with GraphPad Prism Version 9.2.0. Significance between paired groups was assessed using the two-sided Wilcoxon signed rank test, or paired t test. Groups were compared with Kruskal-Wallis test and Dunn´s multiple comparison test, or Mann-Whitney *U* test. For neutralizing antibody titers < 10, a value of 5 was used for the analyses as well as the fold changes shown in **Figures 3A, B**. Ratios of neutralizing antibodies to SARS-CoV-2 IgG were calculated for samples which were positive in both assays. P values <0.05 were considered significant; significance values are indicated as *P < 0.05, ** P < 0.01, *** P < 0.001, **** P < 0.0001.

## Results

### Cohort

The cohort consisted of 26 children diagnosed with SARS-CoV-2 infection who had participated in a population-based SARS-CoV-2 seroprevalence study (27), conducted in the early phase of the pandemic (May-July 2020) before the emergence of VOCs, and 24 SARS-CoV-2 age-matched seronegative children. Age (median [IQR], 13.5 [1.6] vs 12.2 [5.4] years) and sex (13m/13f vs 13m/11f) were similar in both groups (**Table 1**). In the seropositive group, 23% (6/26) had no symptoms, while 77% (20/26) experienced COVID-19 symptoms (**Supplementary** **Table 1**). The first serum sample was collected at a median interval of 3.6 [IQR 0.7] months after symptom onset. Further samples were collected 6, 9, and 12 months after serodiagnosis (visits 1-3). Furthermore, we were able to study the effects of vaccination in infection-naïve children (n=10) compared to those with pre-existing immunity (n=10).

**Table 1:** Characteristics of study cohorts.

	n	Age (yrs)^a^	Sex (%)		Follow-up visits (months post serodiagnosis)^a^		
			m	f	Visit 1	Visit 2	Visit 3
**Total**	50	12.8 [3.5]	26 (52)	24 (48)	5 [0.4]	8 [0]	11 [0.9]
**Seropositive**	26	13.5 [1.6]	13 (50)	13 (50)	5 [0.8]	8 [0]	11 [1]
**Seronegative**	24	12.2 [5.4]	13 (54.2)	11 (45.8)	5 [0]	8 [0]	11 [0.75]

a Values are medians, with IQR in square brackets.

### SARS-CoV-2-Specific Antibody Responses

**Figure 1:**
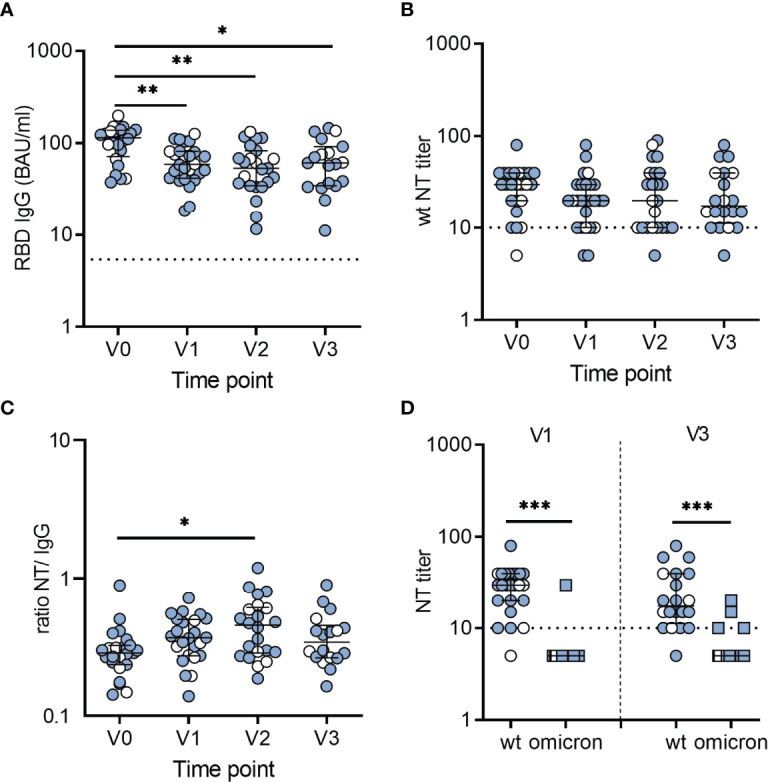
Dynamics and levels of SARS-CoV-2-specific antibodies after infection in children. **(A)** RBD-specific IgG antibodies in BAU/ml. **(B)** Neutralizing antibody titers against an early pandemic virus strain (wildtype, wt). **(C)** Ratios of wt-neutralizing antibody titers and RBD-specific IgG levels. Data for ratios include only samples with a positive result in neutralization assays. Time point V0 represents samples obtained at serodiagnosis; V1, V2, and V3 are follow-up samples obtained 6, 9 and 12 months after serodiagnosis, respectively. **(D)** NT titers against wt and omicron obtained 6 (V1) and 12 months (V3) after serodiagnosis. Bars represent median ± IQR. Blue symbols, symptomatic infection; white symbols, asymptomatic infection. The dashed line indicates the cut-off of the assays. Groups were compared with Kruskal-Wallis test and Dunn´s multiple comparison test (*P < 0.05; **P < 0.01; ***P < 0.001).

Quantification of SARS-CoV-2 spike-specific IgG antibodies was performed with an enzyme-linked immunosorbent assay (ELISA) based on the SARS-CoV-2 RBD. In seropositive children, antibody concentrations declined over 6 months post serodiagnosis, but after this decrease, remained relatively stable between 6 and 12 months (**Figure 1A**). The stratification of children for symptomatic or asymptomatic infection revealed no significant differences in antibody concentrations (labeled in **Figure 1**). To evaluate the kinetics of SARS-CoV-2 spike- and nucleocapsid-specific antibodies, we used a commercial microarray assay based on spike RBD, S1, S2 and nucleocapsid. In line with the ELISA results, antibodies against the RBD were present in all convalescent children and were retained at 12 months after serodiagnosis (**Supplementary** **Figure 1**). In contrast, we observed a decline of nucleocapsid-specific antibodies to undetectable levels in 6 of 26 children (**Supplementary** **Figure 1**).

To analyze the neutralizing activity of antibodies, we performed live-virus neutralization assays using either an isolate from the early pandemic with the D614G mutation (wildtype) or the omicron variant (B.1.1.529) (see Materials and Methods). In seropositive children, neutralizing antibody titers for wildtype were relatively stable over time. As illustrated in **Figure 1B**, neutralizing antibodies decreased to undetectable levels at V1 in two children, one of whom remained undetectable neutralizing antibodies at V2 and V3, and one was lost for follow-up. However, 95% (19 of 20) still having neutralizing antibodies 12 months after serodiagnosis. In agreement with the ELISA results, neutralizing antibodies were not detected in any of the 24 seronegative children (data not shown).

To determine the specific neutralizing activity, we calculated the ratios of neutralizing antibodies to SARS-CoV-2 IgG for the different time points. Notably, the ratios continued to increase over 9 months (V2) and revealed no further change until 12 months (V3) after serodiagnosis, indicating that antibodies improved their neutralizing activity to the original D614G over 9 months (**Figure 1C**). In contrast, there was largely no neutralization of the omicron variant (**Figure 1D**).

### SARS-CoV-2-Specific T Cell Responses

**Figure 2:**
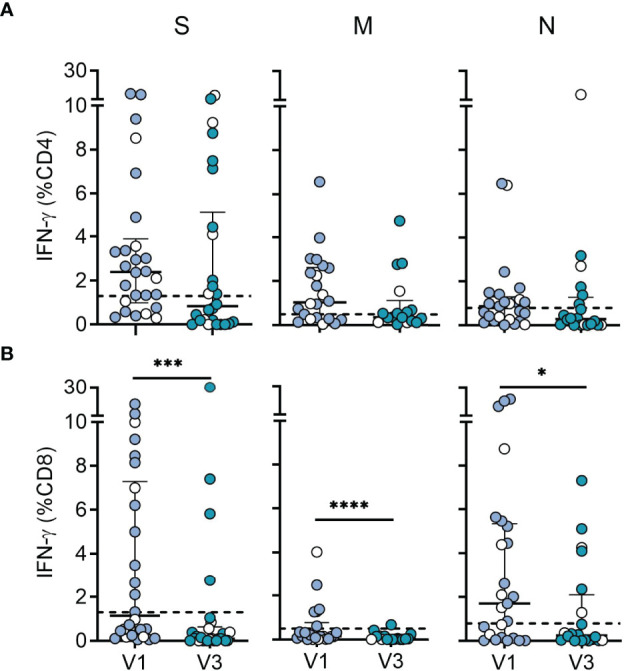
Durability of SARS-CoV-2 specific CD4 and CD8 T cell responses. **(A)** CD4 T cell responses and **(B)** CD8 T cell responses from SARS-CoV-2-seropositive children (n = 26) measured 6 (V1; n = 26) and 12 months (V3; n = 20) after serodiagnosis. Plots show IFN-γ expression levels in response to peptide pools covering the entire sequences of wildtype SARS-CoV-2 spike (S), membrane (M) and nucleocapsid (N) proteins. PBMCs were cultured with spike, membrane and nucleocapsid peptides for 10 days followed by restimulation with the same antigens and analysis of IFN-γ using flow cytometry intracellular cytokine staining. Bars represent median ± IQR. Blue symbols, symptomatic infection; white symbols, asymptomatic infection. The dashed line represents the cut-off for assay positivity. Paired groups were compared with the two sided Wilcoxon signed rank tests (*P < 0.05; ***P < 0.001; ****P < 0.0001).

In addition to antibodies, T cells may contribute to protection by limiting viral dissemination in the host (17). Moreover, CD4 and CD8 T cell responses were recently reported to be less affected by omicron than antibody responses (32–34). We therefore assessed CD4 and CD8 T cell responses in 26 recovered children at 6 and 12 months after serodiagnosis. For sensitive detection of SARS-CoV-2 T cells, antigen-specific T cells were expanded by stimulating PBMCs with peptide pools derived from SARS-CoV-2 wildtype spike, membrane and nucleocapsid for ten days, followed by restimulation with the same antigens and analysis of IFN-γ using flow cytometry intracellular cytokine staining (30) (**Supplementary** **Figure 2**). Among seropositive children, T cell responses to one or more SARS-CoV-2 peptide pools were present in 81% (21 of 26) for CD4 T cells and 85% (22 of 26) for CD8 T cells. CD4 T cells were most frequently directed against spike, followed by membrane and nucleocapsid, while CD8 T cells were mostly directed against nucleocapsid, followed by spike and membrane proteins (**Supplementary** **Figure 3**). In line with these results, CD8 T cell responses directed against nucleocapsid and/or membrane proteins, but not against the spike were observed in 8 children, while only two children mounted nucleocapsid- and/or membrane-specific CD4 T cell responses, but lacked a spike-specific reactivity. There were no significant differences in T cell levels between children with symptomatic or asymptomatic infection (labeled in **Figure 2**). Comparison of responses between 6 and 12 months revealed no significant difference in CD4 T cell levels, while CD8 T cell responses at 12 months were substantially reduced (spike = 10-fold, membrane = 8-fold, nucleocapsid = 7-fold) (**Figure 2**). The specificity of responses at 12 months displayed a similar distribution as at 6 months, with CD4 T cells recognizing spike in 60%, and membrane or nucleocapsid in 30%, respectively, whereas CD8 T cells were directed against nucleocapsid in 30%, followed by spike (20%) and membrane (5%) (**Figure 2** and **Supplementary** **Figure 3**).

**Figure 3:**
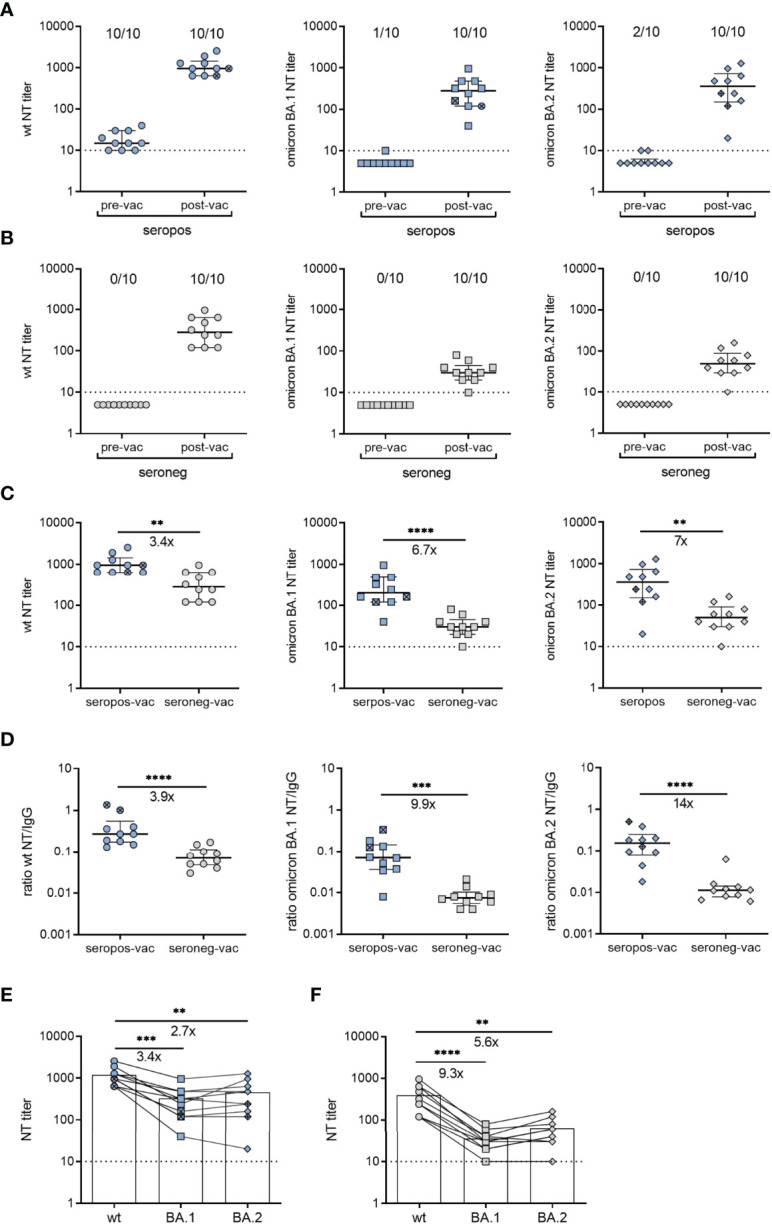
Neutralizing antibodies after vaccination of SARS-CoV-2-recovered and seronegative children. **(A)** Neutralizing antibody titers against an early pandemic virus strain (wildtype, wt), omicron BA.1 or BA.2 variants before and 1.1 [IQR 0.9] months after vaccination (n = 8, BNT162b2; n = 2, Ad26.COV2-S) of children with a confirmed SARS-CoV-2 infection. **(B)** Neutralizing antibody titers against wt strain, omicron BA.1 or BA.2 variants before and 1.3 [IQR 0.6] months after vaccination of seronegative children (n = 10) BNT162b2. **(C)** Post-vaccination neutralizing antibody titers of the two groups against wt strain, or omicron BA.1 or BA.2 variants. **(D)** Ratios of neutralizing antibody titers and RBD-specific IgG values against wt, omicron BA.1 or BA.2 variants **(E)** Postvaccination neutralizing antibody titers against wt, omicron BA.1 or BA.2 from seropositive children (n = 10) and **(F)** seronegative children (n = 10). Samples from the same person are connected by lines. Pre-vac, pre-vaccination; post-vac, post-vaccination; seropos, seropositive; seroneg, seronegative. In panels A and B, the numbers above the plots indicate the proportion of samples that were positive for neutralizing antibodies against the respective variant. Bars represent median ± IQR. Ad26.COV2-S indicated by cross symbol. The dashed line indicates the cut-off of the assays. Groups were compared with the Mann-Whitney *U* test (panels **C**, **D**) and Dunn´s multiple comparison test (panels **E**, **F**) (**P < 0.01; ***P < 0.001; ****P < 0.0001). Fold-differences in panels **(C–F)** are indicated.

We analyzed CD4 and CD8 T cell responses in PBMC samples from 12 of the 24 seronegative children (**Supplementary Figure 4**). In line with previous studies from SARS-CoV-2 unexposed populations (8, 35–40), we observed SARS-CoV-2 reactivity in 17% (2/12) of CD4 and 42% (5/12) of CD8 T cell responses. Consistent with recent data (35), responses in seronegative individuals were focused on spike but lacked reactivity to membrane and nucleocapsid antigens, and are most likely pre-existing cross-reactive T cells induced by endemic common cold coronaviruses or other human viruses (35, 37–43). Together, the data indicate strong CD4 and CD8 T cell responses in recovered individuals with durable CD4 T cell immunity for at least 12 months.

Moreover, we analyzed T cell responses to omicron versus wildtype peptides in PBMCs from 10 of the 26 children at V3. A CD4 T cell response to wildtype versus omicron spike was present in 60% (6/10) versus 50% (5/10), while a CD8 T cell response was detected in 40% (4/10) versus 40% (4/10), respectively (**Supplementary Figures 5A, B**), indicating that there were no significant differences in CD4 or CD8 T cell responses to omicron versus wildtype spike. When comparing the neutralizing activity of antibodies against wildtype and omicron from these children, we found that neutralizing activity to omicron was consistently and significantly lower than to the wildtype strain (**Supplementary Figure 5C**). Overall, the data showed that, unlike neutralizing antibodies, CD4 and CD8 T cell recognition of the omicron spike protein was largely preserved.

### Previous Infection Increases Neutralization Activity Upon BNT162b2 or Ad26.COV2-S Vaccination

To investigate the effect of preexisting immune memory on vaccine responses, we measured antibody titers generated with BNT162b2 or Ad26.COV2-S vaccines in a subgroup of recovered (n=10) and seronegative children (n=10). The samples were collected 1.1 [IQR 0.9] and 1.3 months [IQR 0.6] after receipt of the second dose of BNT162b2 (n=8 recovered; n=10 seronegative) or one dose of Ad26.COV2-S (n=2 recovered). For the comparison of neutralizing activity, we tested pre- and post-vaccination serum samples using live-virus neutralization assays with wildtype and omicron BA.1 and BA.2 variants. Vaccination yielded increased neutralizing antibody titers in both groups (**Figures 3A, B**). However, median titers to SARS-CoV-2 wildtype, omicron BA.1 or BA.2 were 3.4-fold, 6.7-fold or 7-fold higher in recovered than in seronegative children, respectively (**Figure 3C**). Notably, this difference was even more pronounced when we compared specific neutralizing activities, i.e. the ratios of neutralizing antibodies to SARS-CoV-2 IgG. Sera from vaccinated children with pre-existing immunity yielded 3.9-fold, 9.9-fold and 14-fold higher neutralization activities against wildtype, omicron BA.1 and BA.2 than those from seronegative children, respectively (**Figure 3D**). The comparison of individual neutralization profiles between the two groups showed that post-vaccination sera from children with pre-existing immunity efficiently neutralized BA.1 and BA.2, exhibiting 2.7-fold and 3.4-fold differences to the wildtype strain. In contrast, the differences between wildtype and BA.1 or BA.2 were 5.6-fold and 9.3-fold in sera from seronegative children, respectively (**Figures 3E, F**). These results indicate that vaccination of children with preexisting immunity resulted not only in higher neutralizing antibody titers, but also in broader neutralization of VOCs compared to seronegative vaccinees. Analysis of neutralizing antibody titers in non-vaccinated children revealed no differences between samples obtained at V3 (12 months [IQR 0.8]) or 15 months [IQR 1.3] after serodiagnosis (**Supplementary Figures 6A, B**).

## Discussion

In this study, we provide a prospective analysis of the SARS-CoV-2-specific immune response in children after asymptomatic or mild SARS-CoV-2 infection. The data demonstrate that SARS-CoV-2 infection induced long-lived neutralizing antibody responses that substantially improved with subsequent vaccination. Specifically, our data demonstrate that antibodies from recovered vaccinated children exhibited higher neutralizing activity with enhanced cross-neutralization breadth than those induced in infection-naïve vaccinees.

Our finding that infection-induced antibodies against the spike exhibited stabile dynamics over 12 months adds to recent studies exploring long-term immunity in children (8–11, 13). However, in two children, neutralizing antibodies decreased to undetectable levels at 6 months after serodiagnosis. In contrast, nucleocapsid-specific antibodies decreased to undetectable levels in around one-fourth of the children, which confirms previous findings (10, 13) and is consistent with the longer half-life of spike-specific compared with nucleocapsid-specific IgG responses (44).

In addition to durable spike-specific antibody responses, we detected robust CD4 and CD8 T cell responses in recovered children, with persistent CD4 T cell immunity for at least 12 months. Furthermore, our data showed that while antibodies generated after infection did not effectively cross-neutralize the omicron variant, the SARS-CoV-2 spike-specific T cell response was largely preserved against omicron. It is important to note that the sample size in the present study was small, however the findings are consistent with results obtained from a number of studies with adults, indicating that SARS-CoV-2 T cell responses are less affected by viral immune escape (32–34).

Finally, when comparing vaccine-induced responses from recovered and infection-naïve children, we found that those with preexisting immunity had higher neutralizing antibody titers. It was also remarkable that vaccination of recovered children further enhanced the specific neutralizing activity to omicron BA.1 and BA.2 variants relative to an ancestral D614G strain (**Figure 3**). Our data are thus in line with recent studies in adults that showed efficient neutralization of VOCs, including omicron, after encountering the spike during infection followed by vaccination or vice versa (45, 46). A further aspect that has to be considered is the long interval between infection and vaccination (12-15 months) in our cohort during which the neutralizing activity may have improved and contributed to the observed high neutralization titers. This is consistent with recent studies showing that antibodies evolve in convalescent patients due to affinity maturation, which results in an increase in the breadth and neutralizing potency of antibodies against SARS-CoV-2 (47–50). It will be of importance to elucidate whether sustained immunity evolves in a similar manner in naïve children who received vaccination, and whether efficient neutralization of VOCs occurs upon additional boosting. Overall, our data provide new insights into the durability and breadth of immune memory induced by SARS-CoV-2 infection in children. By demonstrating the immunological benefits of boosting infection-induced immunity, these findings may serve as guides for the ongoing development of vaccine strategies for children.

## Data Availability Statement

The original contributions presented in the study are included in the article/**Supplementary Material**. Further inquiries can be directed to the corresponding authors.

## Ethics Statement

The studies involving human participants were reviewed and approved by the ethics committee of the Medical University of Vienna, Austria. Written informed consent to participate in this study was provided by the participants’ legal guardian/next of kin.

## Author Contributions

JA, ZS, JS, and KSt designed the study and wrote the manuscript. JA and KSt designed experimental approaches. JA, KSt, JS, MM, JC, AP, LW, and KSc performed, analyzed, and interpreted the results. JS, JH, AS, KSc, SK, and VD collected blood samples and provided clinical data. Every author has read, edited and approved the final manuscript.

## Funding

The study was supported by Medical-scientific funds of the Mayor of the federal capital Vienna [grants Covid003, Covid028 and 21205].

## Conflict of Interest

The authors declare that the research was conducted in the absence of any commercial or financial relationships that could be construed as a potential conflict of interest.

## Publisher’s Note

All claims expressed in this article are solely those of the authors and do not necessarily represent those of their affiliated organizations, or those of the publisher, the editors and the reviewers. Any product that may be evaluated in this article, or claim that may be made by its manufacturer, is not guaranteed or endorsed by the publisher.
