# Supplementary material for: Long-Lived Immunity in SARS-CoV-2-Recovered Children and Its Neutralizing Capacity Against Omicron
Source: Front Immunol. 2022 May 17;13:882456. doi: 10.3389/fimmu.2022.882456 (PMC9157051; doi:10.3389/fimmu.2022.882456)
Supplement: Supplementary file 1 [file DataSheet_1.pdf]

Supplementary Material

Long-lived immunity in SARS-CoV-2-recovered children and its neutralizing capacity against omicron

Justyna Sieber[1,2], Margareta Mayer[3], Klara Schmidthaler[1], Sonja Kopanja[1], Jeremy V. Camp[3], Amelie Popovitsch[3], Varsha Dwivedi[1], Jakub Hoz[1], Anja Schoof[1], Lukas Weseslindtner[3], Zsolt Szépfalusi[1*] , Karin Stiasny[3*], Judith H. Aberle[3*]

[1]Division of Pediatric Pulmonology, Allergy and Endocrinology, Department of Pediatrics and Adolescent Medicine, Comprehensive Center of Pediatrics, Medical University of Vienna, Vienna Austria
[2]Department of Clinical Immunology, Wroclaw Medical University, Wroclaw, Poland

[3]Center for Virology, Medical University of Vienna, Vienna, Austria

[*]Correspondence

Judith H. Aberle: judith.aberle@meduniwien.ac.at

Karin Stiasny: karin.stiasny@meduniwien.ac.at

Zsolt Szépfalusi: zsolt.szepfalusi@meduniwien.ac.at

Supplementary Table S1. Clinical characteristics of recovered children

#Patient	Symptoms
P01	Asymptomatic
P02	Headache, rash
P03	Headache
P04	Headache, fever, fatigue, sore throat, dyspnea, anosmia, ageusia, muscle and joint aches
P05	Headache, fever, fatigue, sore throat, dyspnea, muscle and joint aches, cough, nausea, stomachache, diarrhea
P06	Headache, subfebrile, fatigue
P07	Asymptomatic
P08	Headache, fever, fatigue, sore throat, muscle and joint aches
P09	Headache, fever, fatigue, sore throat
P10	Headache, fever, fatigue, sore throat, cough, stomachache
P11	Asymptomatic
P12	Headache, subfebrile, fatigue
P13	Headache, fever, fatigue, sore throat, cough, muscle and joint aches, rhinitis
P14	Asymptomatic
P15	Headache, anosmia, ageusia, stomachache
P16	Headache, sore throat, cough, dyspnea
P17	Headache, fatigue, anosmia, ageusia
P18	Headache, fever, fatigue, anosmia, ageusia
P19	Asymptomatic
P20	Asymptomatic
P21	Anosmia, ageusia
P22	Anosmia, ageusia, sore throat, rhinitis
P23	Headache, fatigue, cough, anosmia, ageusia, rash
P24	Headache, fatigue, anosmia, ageusia, dyspnea, muscle and joint aches
P25	Earache, rhinitis
P26	Anosmia, ageusia

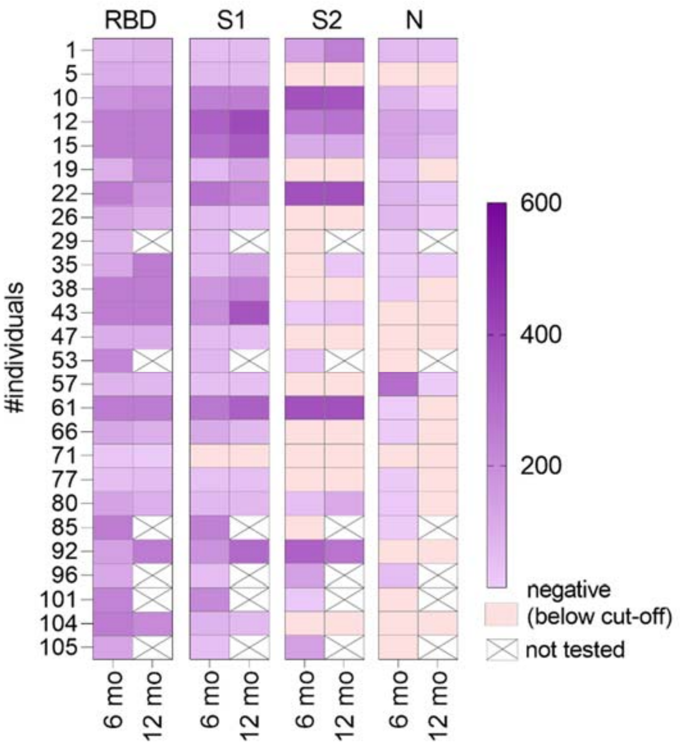

Supplementary Figure 1. SARS-CoV-2-specific IgG antibodies at 6 and 12 months after infection. Heatmap indicating IgG antibody levels in BAU/ml against SARS-CoV-2 receptor binding domain (RBD), spike S1, S2 and nucleocapsid (N) antigen in recovered children at 6 and 12 months after serodiagnosis.

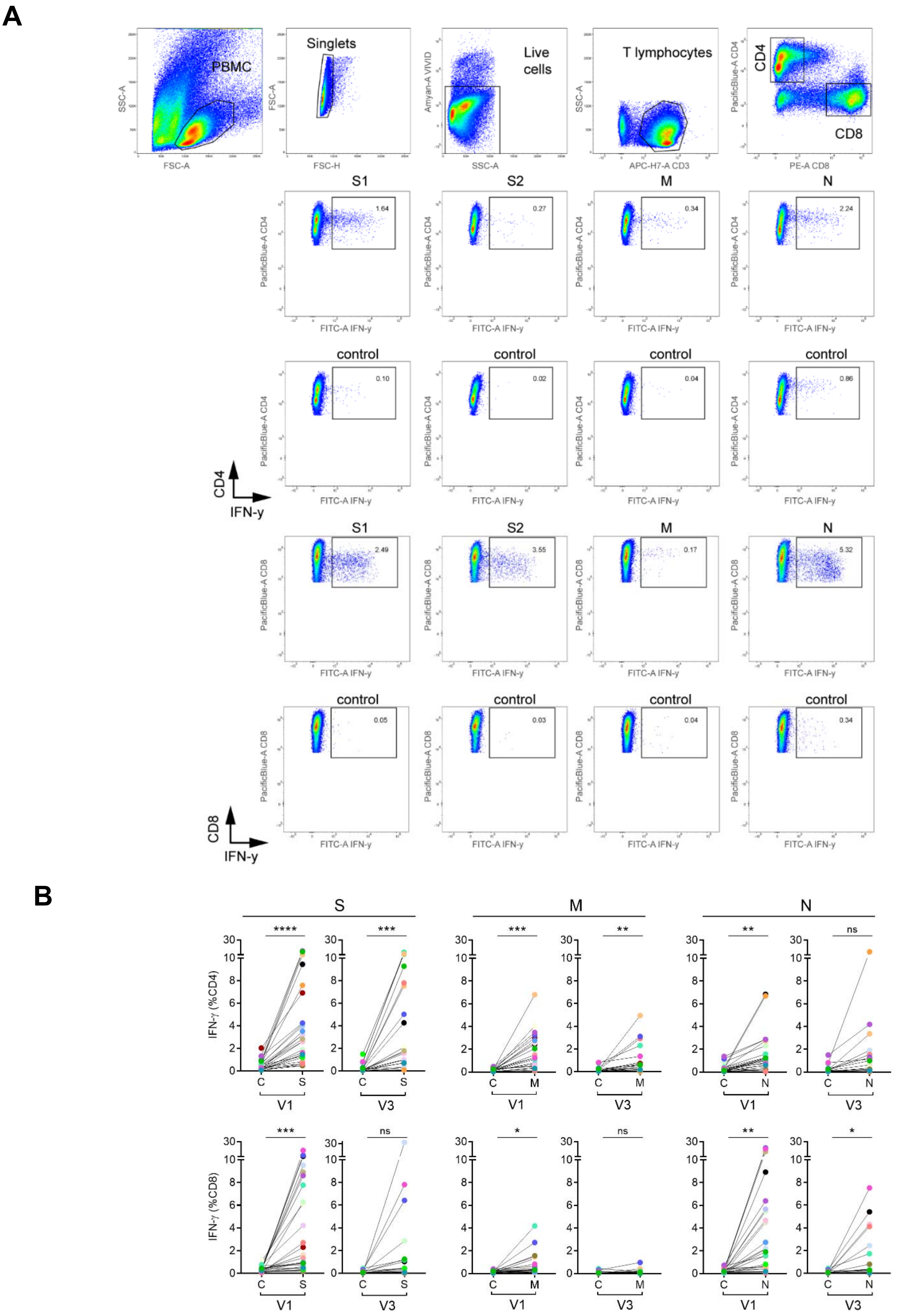
A
PBMC
Singlets
Live cells
T lymphocytes
CD4
CD8
SSC-A
FSC-A
FSC-H
Amyan-A VIVID
APC-H7-A CD3
PacificBlue-A CD4
PE-A CD8
S1
S2
M
N
1.64
0.27
0.34
2.24
control
0.10
0.02
0.04
0.86
FITC-A IFN-y
PacificBlue-A CD8
2.49
3.55
0.17
5.32
0.05
0.03
0.04
0.34
CD4
IFN-y
CD8
IFN-y
B
S
M
N
****
***
**
ns
*
IFN-γ (%CD4)
IFN-γ (%CD8)
C
V1
V3

Supplementary Figure 2. Gating strategy and analysis of SARS-CoV-2-specific T cell response. (A) CD4 and CD8 T cell reactivity to SARS-CoV-2 peptide pools spike (S1, S2) membrane (M) and nucleocapsid (N) shown for one SARS-CoV-2 seropositive individual. PBMCs were cultured with S1, S2, M or N for 10 days followed by restimulation with the same antigens or no antigen (control), and analyzed for intracellular staining IFN-γ using flow cytometry. Gates were set in the samples with no antigen stimulation (control). (B) Paired analysis of IFN-γ expression levels in CD4 (upper panels) and CD8 T cell responses (lower panels) between the negative control (C) and antigen-specific stimulation with S (sum of S1 and S2; n=26), M (n=22) or N (n=25) at 6 months (V1) and 12 months (V3, n=20 for S, M and N, respectively) after serodiagnosis. Paired t test was used to compare T cell responses upon stimulation by SARS-CoV-2 peptide pools versus controls (* $P < 0.05$; ** $P < 0.01$; *** $P < 0.001$; **** $P < 0.0001$; ns >0.05).

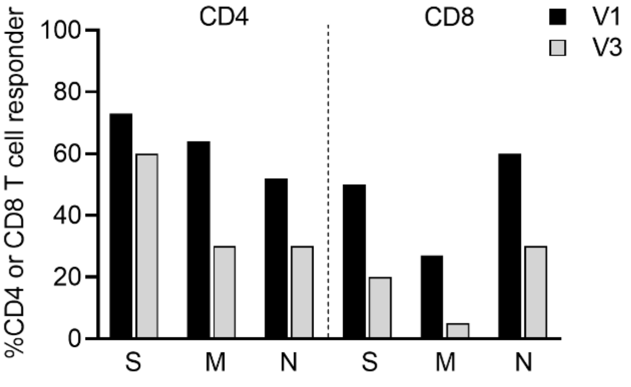

Supplementary Figure 3. Percentage CD4 and CD8 T cell responses in SARS-CoV-2 seropositive children. Bars indicate the proportions of seropositive children who demonstrated a CD4 or CD8 T cell responses to wildtype SARS-CoV-2 spike S (n=26), membrane M (n=22) or nucleocapsid N (n=25) proteins at 6 months (V1) and 12 months (V3; n=20 for S, M and N, respectively) after serodiagnosis.

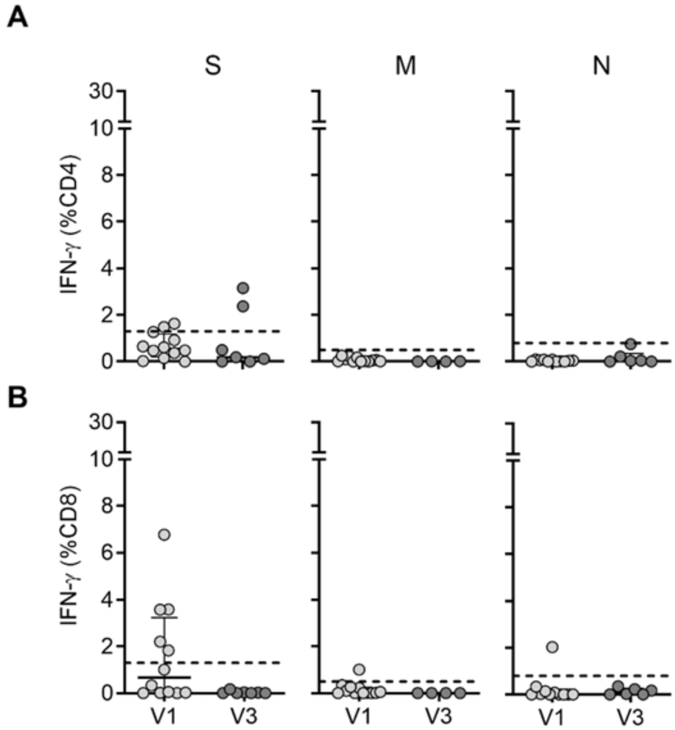

Supplementary Figure 4. T cell response in age-matched seronegative controls. (A) CD4 T cell responses and (B) CD8 T cell responses from seronegative controls (n=12) measured 6 (V1) and 12 months (V3) after serodiagnosis. Plots show IFN-γ expression levels in response to peptide pools covering the entire sequences of wildtype SARS-CoV-2 spike (S), membrane (M) and nucleocapsid (N) proteins. Bars represent median ± IQR. The dashed lines represent cut-off values for the different SARS-CoV-2 peptide pools (S, M and N).

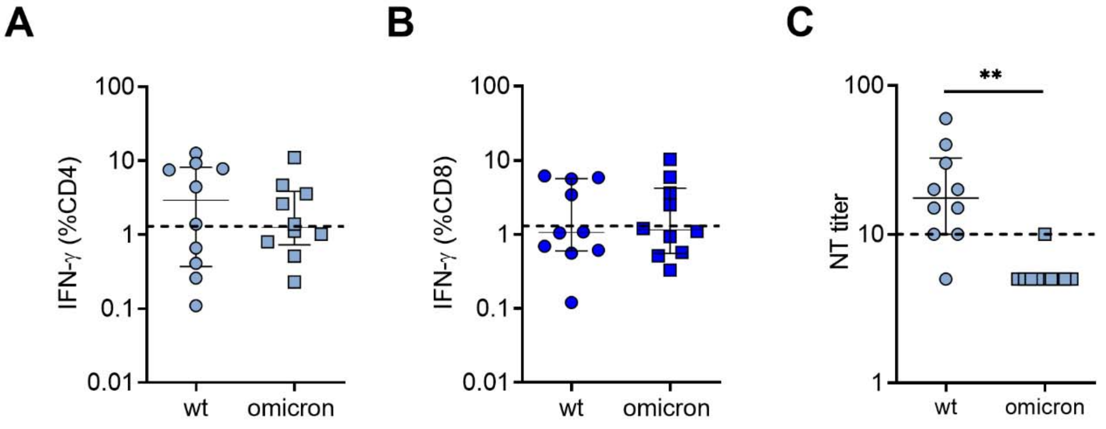

Supplementary Figure 5. T cell response to SARS-CoV-2 wildtype and omicron spike in SARS-CoV-2 recovered children. PBMCs at 12 months (V3) after serodiagnosis of n=10 recovered children were cultured with SARS-CoV-2 wildtype (wt) and omicron BA.1 spike peptide pools for 10 days, followed by restimulation with the same antigens or no antigen and analyzed for intracellular IFN-γ staining using flow cytometry. (A) CD4 and (B) CD8 T cell responses to SARS-CoV-2 wt and omicron BA.1 spike peptide pools. (C) Neutralizing antibody titers against wt and omicron BA.1. Bars represent median ± IQR. Dashed lines indicate the cut-offs of the assays. Differences between wt and omicron were calculated using two sided Wilcoxon signed rank tests (** $P < 0.01$).

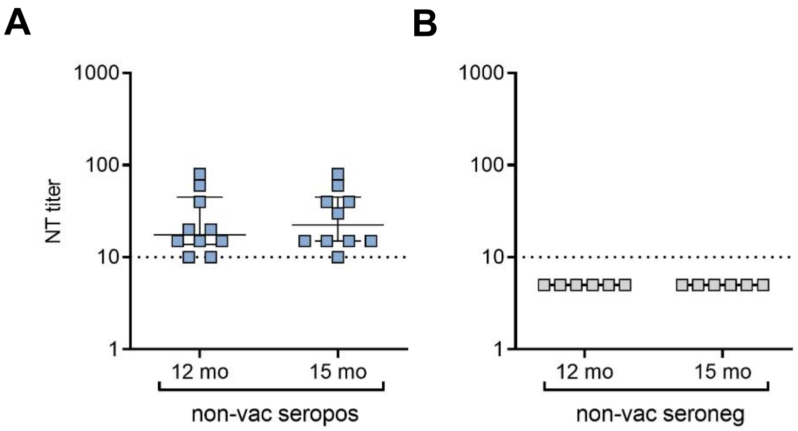

Supplementary Figure 6. Neutralizing antibodies in non-vaccinated SARS-CoV-2-recovered and seronegative children. (A) Neutralizing antibody titers in serum samples obtained at 12 months and 15 months after serodiagnosis in non-vaccinated children with a confirmed SARS-CoV-2 infection (n=10) or (B) seronegative children (n=6); non-vac, non-vaccinated; seropos, seropositive; seroneg, seronegative.
